# Interleukin-28B dampens protease-induced lung inflammation via IL-25 and TSLP inhibition in epithelial cells

**DOI:** 10.1038/s41598-020-77844-y

**Published:** 2020-12-01

**Authors:** Bailing Yan, Jinying Gao, Jia Guo, Dong Yang, Dan Li

**Affiliations:** 1 Department of Respiratory Medicine, The First Hospital of Jilin University, 1Xinmin Street, Changchun, 130021 Jilin Province People’s Republic of China; 2 Department of Gastroenterology, The First Hospital of Jilin University, 1Xinmin Street, Changchun, 130021 Jilin Province People’s Republic of China

**Keywords:** Medical research, Innate immunity

## Abstract

Asthma is a chronic respiratory disease with high heterogeneity in human. Different mouse models have been applied for investigation of pathogenesis and treatment of asthma, which target on different cells, receptors and pathways. Interleukin (IL-) 28B, a member of λ-interferons, have been shown to play a protective role in OVA-induced asthma, which is antigen-specific and adaptive immune system dominant. However, the roles of IL-28B in protease-induced asthma, an adaptive immune system independent asthma, are still unclear. Here, we used plant-derived cysteine protease, papain to induce asthma in mice and found that IL-28B was capable of alleviating papain-induced asthma. Papain challenge lead to activation of epithelial cells and production of alarmin, such as IL-25 and thymic stromal lymphopoietin and IL-28B treatment down-regulated their production. Further mechanism was proved to be that IL-28B inhibited the phosphorylation of Erk in epithelial cells via interaction with their receptors. Our results reveal a protective role of IL-28B via regulation of epithelial cells in protease induced asthma.

## Introduction

Asthma is a chronic pulmonary disease characterized by airway hyper-responsiveness (AHR), inflammatory immune response, mucus overproduction, airway remodeling and airflow limitation. This disease affects about 300 million populations worldwide^1^. The pathogenesis of asthma is complex and not well understood. In human, asthma is heterogeneous and able to divide into several subtypes based on disease onset, pathogenesis, clinical parameters and physiological features^2^. To mimic and cover biological features of the heterogeneity of human asthma, different mouse models have been developed based on different signaling pathways and different cell types^3^. For example, ovalbumin(OVA)/alum induced asthma is triggered by NOD-like receptor pyrin domain-containing protein 3 (NLRP3)/interleukin (IL)-1β activation by phagocytes and immunoglobulin E (IgE) production by B cells, which highly depends on adaptive immune system^4,5^. However, plant-derived cysteine protease, papain induces asthma in mice initiated by activation of protease-activated receptor (PAR) signaling pathway and the subsequent production of IL-33, IL-25 and thymic stromal lymphopoietin (TSLP) from airway epithelial cells, followed by secretion of IL-5 and IL-13 by lung group 2 innate lymphoid cells (ILC2s)^3,6^. Different from OVA/alum induced asthma, papain can induce asthma in the absence of adaptive immune system, which resemble an “innate-like” asthma. Other models such as HDM, an animal-derived counterpart of papain, and Alternaria althernata have also been applied^7^. Notably, the epithelial cells of airways serve as the first line of defense against inhaled allergens. However, the innate regulation of the epithelial cells in response to different allergens are still nuclear.

IL-28 is a family of cytokines that belongs to interferons termed as λ-interferons or type III interferons (IFN). This family includes IL-28A(IFN-λ2), IL-28B(IFN-λ3) and IL-29(IFN-λ1) in human and IL-28A and IL-28B in mice. IL-28A, IL-28B and IL-29 share a unique heterodimeric receptor IFNLR consisting of IL-28Rα and IL-10R, which is mainly expressed by epithelial cells, dendritic cells (DCs) and macrophages^8–10^. Data have revealed the roles of IL-28B and IL-28A in asthma^11–13^. Particularly, our studies have previously shown that IL-28B dampens airway inflammation through upregulation of IFN-γ from natural killer cells in OVA/alum induced asthma model^14^. However, the roles of IL-28B in papain induced asthma and the underlie mechanism are still unknown.

Here, we established papain induced asthma model in mice and found that IL-28B could also alleviated papain-induced airway inflammation. Airway derived IL-25 and TSLP were significantly reduced after IL-28B treatment. Further, the results revealed that IL-28B regulates IL-25 and TSLP expression in epithelial cells via Erk signaling pathway. These data suggested that IL-28B dampens protease-induced lung inflammation, mainly via inhibition of IL-25 and TSLP in epithelial cells.

## Materials and methods

### Mice and asthma model

Male C57BL/6 mice (8–10 weeks) were purchased from Shanghai SLAC Laboratory Animal center, Chinese Academy Science (Shanghai, China). All experimental protocols were approved by the Institutional Ethics Committee for Animal Use in Research of Jilin University and the methods were carried out in accordance with Animal Care guidelines of Jilin University. All mice were housed in under humidity- and temperature-controlled specific pathogen-free condition. For papain-induced lung inflammation, mice were anesthetized and exposed intranasally to 20 μg papain (Sigma-Aldrich, St. Louis, MO, USA) in 40 μl PBS once a day for 6 days. One day after the last challenge, bronchoalveolar lavage was performed and the lung tissues were harvested.

### Recombinant plive vectors and hydrodynamic injection

The plive vector (Mirus Bio, Madison, WI) is a kind of liver-specific transgene expression vector that was described previous. Recombinant plive vector expressing IL-28B (plive-IL-28B) was kindly provided by Dr. Yanshi Wang (The First Affiliated Hospital of USTC). The vector was purified using the Endo-Free Maxi plasmid kit (Macherey–Nagel, Duren, Germany). 20 μg of purified vector dissolved in PBS in a volume equivalent to 8% of the mouse body weight was injected via tail veins within 5 s on day 0 as indicated in the experimental protocol.

### Airway hyper-responsiveness

The airway resistance was measured in anesthetized mice one day after the last challenge using a respiratory function instrument (AniRes2005, BioMedical Supply Co., Ltd., Beijing, China) according to the manufacturer’s instructions. Briefly, the mice were connected to a computer-controlled ventilator via a tracheal cannula. They were placed supine inside a plexiglass whole-body plethysmograph. The pressure changes in the plethysmographic chamber were measured through a port in the connecting tube with a pressure transducer. The mice were mechanically ventilated with a tidal volume of 5 ml/kg at a rate of 150 breaths/min with a positive end-expiratory pressure. The mice were initially challenged with aerosol saline followed by challenge with increasing concentrations of methacholine (0, 5, 10, 25 and 40 mg/ml; Sigma-Aldrich) for 10 s at each dose.

### Collection of bronchoalveolar lavage fluid and isolation of lung immune cells

Collection of bronchoalveolar lavage fluid for cytokine analysis and cell subset analysis was performed as previously described^5^. Briefly, the whole lung was perfused through the trachea with 1 ml of PBS containing 5 mM of EDTA for 4 times. The bronchoalveolar lavage fluid (BALF) was collected and cell pellets were pooled. Isolation and sorting of the lung immune cells were performed as previously described^5,15^. To obtain single-lung-cell suspensions, the lungs were minced and digested in RPMI-1640 medium containing 0.1% collagenase I (Sigma-Aldrich), 2 μg/ml DNase I (Sigma-Aldrich) and 10% fetal bovine serum (Gibco, Paisley, Scotland) for 60 min at 37 °C. After filtration through a 100 μm Cell Strainer, the medium was centrifuged and the red blood cells (RBCs) were removed by RBC lysis buffer (BioLegend, San Diego, CA, USA). The cells were washed by FACS buffer and collected for Flow Cytometer. To purify different immune cell subsets, we sorted Neutrophils (CD45^+^ CD11b^+^Ly6G^hi^), AMs (CD45^+^F4/80^hi^CD11c^hi^), DC(CD45^+^F4/80^−^MHC-II^hi^CD11c^hi^), T(CD45^+^CD3^+^CD19^−^CD11b^−^) and B(CD45^+^CD3^−^CD19^+^ CD11b^−^) cells by BD Aria II Cell Sorter (BD Biosciences, San Diego, CA, USA).

### Cell culture

Mouse lung epithelial cell line (MLE-12) and primary lung epithelial cells were cultured with 200 ng/ml papain. After 2 h, IL-28B (100 ng/ml) was added and cocultured for 24 h. Then the supernatant and cells were harvested for analysis.

### Isolation of alveolar epithelial cells

Isolation of alveolar epithelial cells were performed as previously described^5,15^ with modification. The lung lobes were dissected away, digested in a 50 ml conical tube with 5 ml of complete DMEM containing 50 U/ml of Dispase II (Sigma-Aldrich) and gently shaken at room temperature for 45 min. The suspension was passed through 70 μm Cell Strainers, spun down and resuspended in complete DMEM. After flow antibodies staining, the cells were washed and epithelial cells were sorted as lineage (Lin) markers (CD45, CD16/32, CD31, Ter119) negative and EpCAM positive live cells by BD Aria II Cell Sorter.

### Flow cytometry

After the blockade of the Fc receptor by rat serum, single-cell suspensions were incubated with the fluorescently labelled monoclonal antibodies at 4 °C for 30 min in PBS containing 0.1% sodium azide and 1% bovine serum albumin, and then washed twice followed analyzed by flow cytometry (LSR II; BD Biosciences) using FLOWJO 10 software (BD Biosciences). The anti-mouse monoclonal antibodies used for flow cytometry were as follows: Ter119 (clone Ter119), CD45(30-F11), CD3(17A2), Ly6G(1A8), CD19(6D5), F4/80(BM8), CD11c(HL3), CD11b(M1/70), SiglecF(E50-2440), MHC-II(M5/114.15.2) and EpCAM(G8.8), CD16/32 (2.4G2), CD31(MEC13.3). Lineage markers: CD45, CD16/32, CD31, Ter119. All antibodies and isotype controls were purchased form BD Biosciences (Franklin Lakes, NJ, USA), R&D Systems (Minneapolis, MN, USA), or eBioscience (San Diego, CA, USA). Cell phenotypes were identified as previously described^14^.

### Histology

Lung tissues were fixed in 10% (v/v) formalin for > 24 h and embedded in paraffin. Sections (thickness, 5 μm) were stained with periodic acid–Schiff's solution (Solarbio, Beijing, China). More than 6 sections from the lungs were selected randomly to determine inflammatory injury, and the reviewer was blinded to the treatments. A semi-quantitative scoring system was used to evaluate the degree of the inflammation: Both peribronchiolar and perivascular inflammation were scored giving a maximum score of 8 as follows: 0, normal; 1, few cells; 2, a ring of inflammatory cells one cell layer deep; 3, a ring of inflammatory cells two to four cells deep; and 4, a ring of inflammatory cells of more than four cells deep.

### Real-time PCR

For real-time PCR, 10–50 mg of lung tissues or 10^5^–10^6^ of purified cells was used for RNA extraction. Total RNA was extracted using TRIzol Reagent (Invitrogen, Carlsbad, CA, USA). cDNA was generated using oligo-dT, random hexamers, and Super-Script RT II (Invitrogen). Then, expression of indicated genes was analyzed according to the instructions of the SYBR Premix Ex Taq kit (TaKaRa Bio, Kusatsu, Japan) and quantified using the ^ΔΔ^Ct method. The primers were as followed (all for mice):

- gapdh-F 5′-TACCCCCAATGTGTCCGTC-3′,
- gapdh-R 5′-AAGAGTGGGAGTTGCTGTTGAAG-3′,
- tslp-F 5′-GGAGATTTGAAAGGGGCTAAG-3′,
- tslp-R 5′-TGGGCAGTGGTCATTGAG-3′,
- il-25-F 5′-TGGCAATGATCGTGGGAACC-3′,
- il-25-R 5′-GAGAGATGGCCCTGCTGTTGA-3′,
- ifn-γ-F 5′-CGGCACAGTCATTGAAAGCCTA -3′,
- ifn-γ-R 5′-GTTGCTGATGGCCTGATTGTC-3′,
- il-10-F 5′-CACAAAGCAGCCTTGCAGAA -3′,
- il-10-R 5′-AGAGCAGGCAGCATAGCAGTG-3′,
- il-28ra(ifnlr1)-F 5′- AAGTTCAAAGGACGAGTACAGG-3′,
- il-28ra(ifnlr1)-R 5′-GTACTTCAGTTCCAGCGACG -3′

All primers were synthesized by Sangon Biotech (Shanghai, China).

### ELISA

The supernatant of cell culture was frozen at − 80 °C before use. The concentrations of IL-25 and TSLP were determined using the Mouse ELISA kits (Dakewe Biotech, Shenzhen, China. eBioscience), following the manufacturer’s instructions.

### Western blot

The lung tissues or MLE-12 cells were lysed in lysis buffer (Cell Signaling, Boston, MA, USA) and cell debris was removed the supernatant was boiled for 5 min in loading buffer (GenScript, Nanjing, China) for 20 min. An equal amount of proteins was subjected to SDS-PAGE before blotting onto a PVDF membrane. The membrane was blocked with 5% skim milk and probed with anti-mouse β-actin, phospho-ERK, total ERK, IL-25, IL-10, TSLP, IFN-γ specific antibodies (R&D Systems and Cell Signaling) at 4℃ overnight. After washing, the membranes were incubated for 1 h with secondary antibody coupled to horseradish peroxidase and detected using an ECL chemiluminescent detection system (GenScript, Nanjing, China), following the manufacturer’s instructions.

### Statistical analysis

All data were expressed as mean ± SEM and analyzed using the Student’s two-tailed t-test or one-way analysis of variance (ANOVA) test followed by LSD’s multiple comparison test. A value of *P* < 0.05 was considered statistically significant.

## Results

### IL-28B alleviated papain-induced airway hyper-responsiveness and inflammation in mice

**Figure 1:**
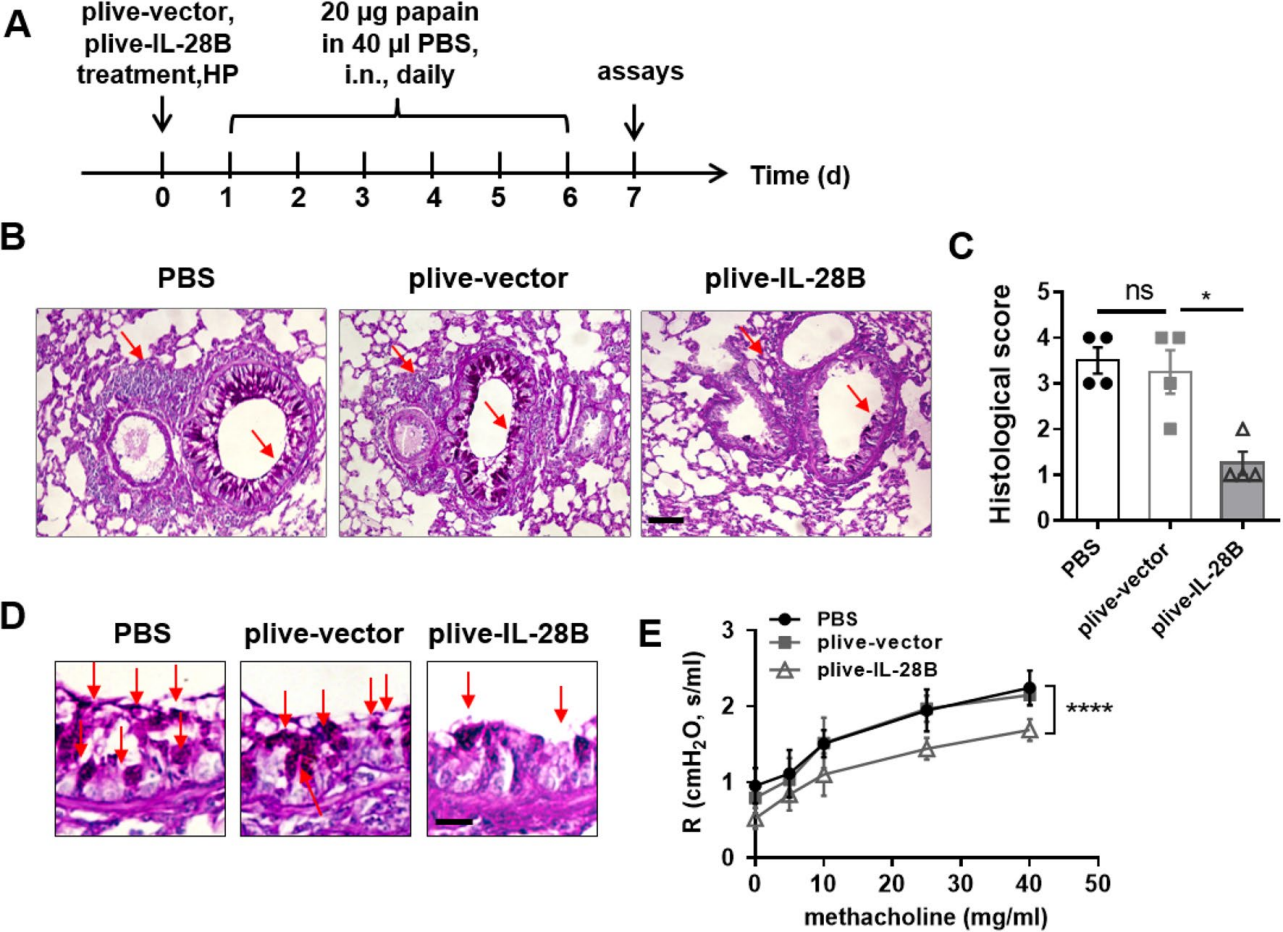
IL-28B alleviated papain-induced airway injury in mice. (**A**) Work flow for IL-28B treatment in papain-induced asthmatic mice. Briefly, mice were injected (h.p.) with plive-IL-28B or plive-vector or PBS on day 0 and were subsequently challenged with papain for 6 consecutive days. All mice were assayed on day 7. (**B**) Histologic sections of lung from PBS-, plive-vector- or plive-IL-28B- treated asthmatic mice were stained with Periodic Acid-Schiff (PAS) and analyzed by light microscopy. Scale bar = 100 μm. (**C**) Histological scores for assessment of lung injury were shown. (**D**) PAS staining for mucus production in lung epithelium from each group were shown. Scale bar = 50 μm. (**E**) Airway resistance was measured in response to increasing doses of aerosolized methacholine in mice (two-way ANOVA comparing plive-vector-treated and plive-IL-28B-treated mice). Data are representative of three or more independent experiments with 4 mice per group. Data are the mean ± SEM. *P < 0.05, ****P < 0.0001, ns = not significant. (Two-way ANOVA for **E** and student’s t-test for **C**). h.p., hydrodynamic injection; i.n., intranasally injection.

To investigate the role of IL-28B in papain-induced airway hyper-responsiveness, we employ the plasmid plive–IL-28B in mice, which supported over-expression of IL-28B for more than two weeks after hydrodynamic tail vein injections^14,16^. The mice were treated with plive-IL-28B plasmids or plive-vector plasmids or PBS followed by 6-consecutive-day of papain challenge in airway (Fig. 1A). The histological staining revealed that injection of plive-IL-28B but not plive-vector could significantly inhibit papain induced inflammatory cell infiltration to lung tissues and also airway damage (Fig. 1B,C). And plive-IL-28B markedly reduced mucus over-production in airway (Fig. 1D). Consistently, airway resistance, which is canonical feature of asthma, was lower in plive-treated mice compared with plive-vector- or PBS-treated mice (Fig. 1E and Supplementary Fig. 1). These data suggested that IL-28B was capable of alleviating papain-induced airway hyper-responsiveness (AHR) in mice.

**Figure 2:**
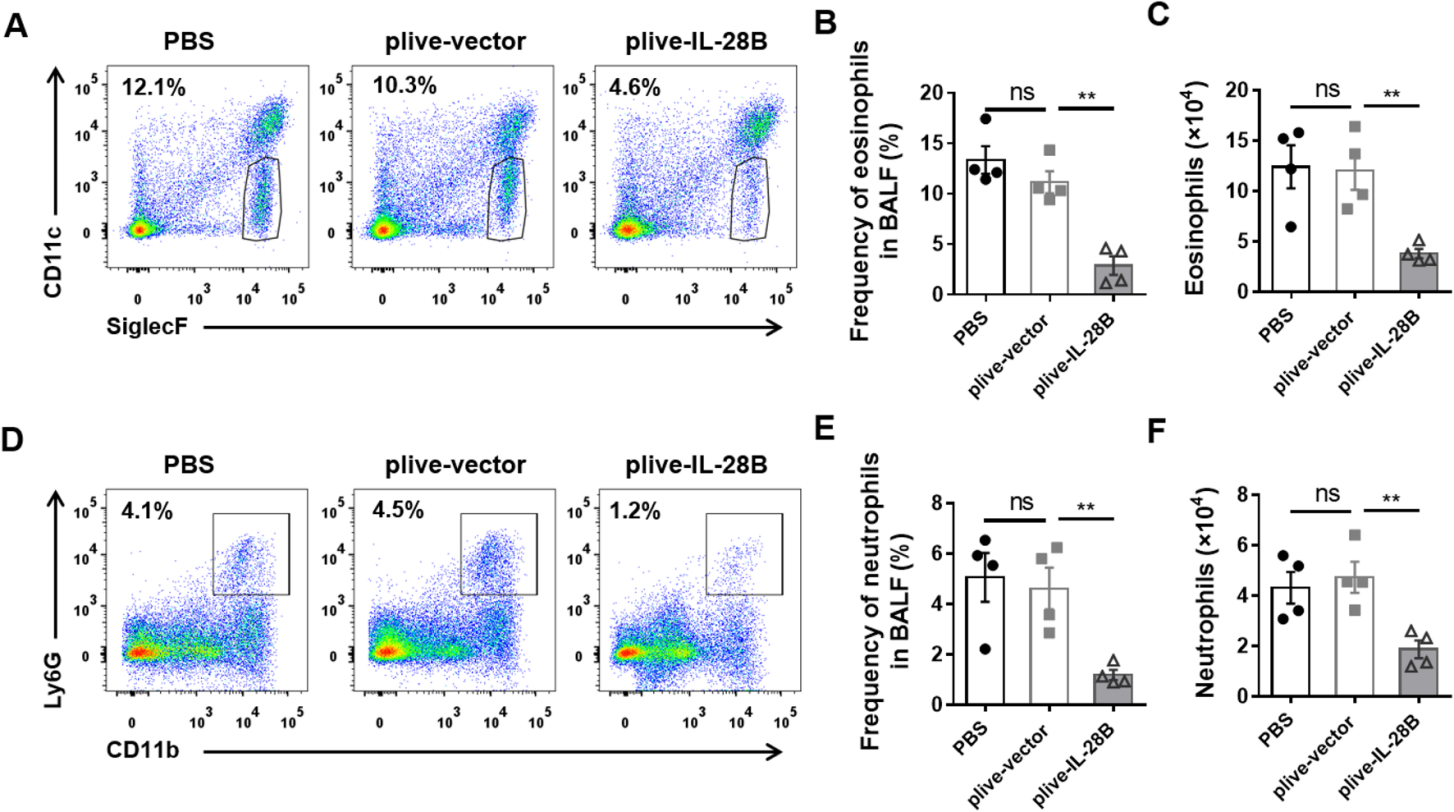
IL-28B restricted inflammatory cell infiltration in the lung. Mice were injected (h.p.) with plive-IL-28B or plive-vector or PBS on day 0 and were subsequently challenged (i.n.) with papain for 6 consecutive days. All mice were harvested bronchoalveolar lavage fluid (BALF) were collected on day 7. The cells in BALF were analyzed by flow cytometry (FACS). (**A**) Dot plots for eosinophils were analyzed by FACS. Eosinophils were gated as CD45^+^SiglecF^+^CD11c^−^. (**B**) The frequency of eosinophils was shown. (**C**) The number of total cells was counted and the eosinophils were calculated. (**D**) Dot plots for neutrophils were analyzed by FACS. Neutrophils were gated as CD45^+^CD11b^+^Ly6G^+^. (**E**) The frequency of neutrophils was shown. (**F**) The number of neutrophils was calculated. Data are representative of three or more independent experiments with ≥ 4 mice per group. Data are the mean ± SEM. **P < 0.01, ns = not significant. h.p., hydrodynamic injection; i.n., intranasally injection.

To further dissect which type of cells were influenced by IL-28B, we collected bronchoalveolar lavage fluid (BALF) and analyzed the cellular components by flow cytometry. We found that the frequency and number of eosinophils (CD45^+^SiglecF^+^CD11c^−^) were much lower in plive-IL-28B treated mice compared with plive-vector or PBS treated mice (Fig. 2A–C). Neutrophils were another inflammatory cell that cause tissue damage. The results showed that the frequency and number of neutrophils (CD45^+^CD11b^+^Ly6G^+^) were also reduced in plive-IL-28B treated mice compared with plive-vector or PBS treated mice (Fig. 2D–F). Collectively, these dates indicated that treatment of plive-IL-28B could alleviate papain-induced AHR and infiltration of inflammatory cells including eosinophils and neutrophils, suggesting a protective role of IL-28B in papain-induced asthma model.

### IL-28B reduced papain-induced production of IL-25 and TSLP

**Figure 3:**
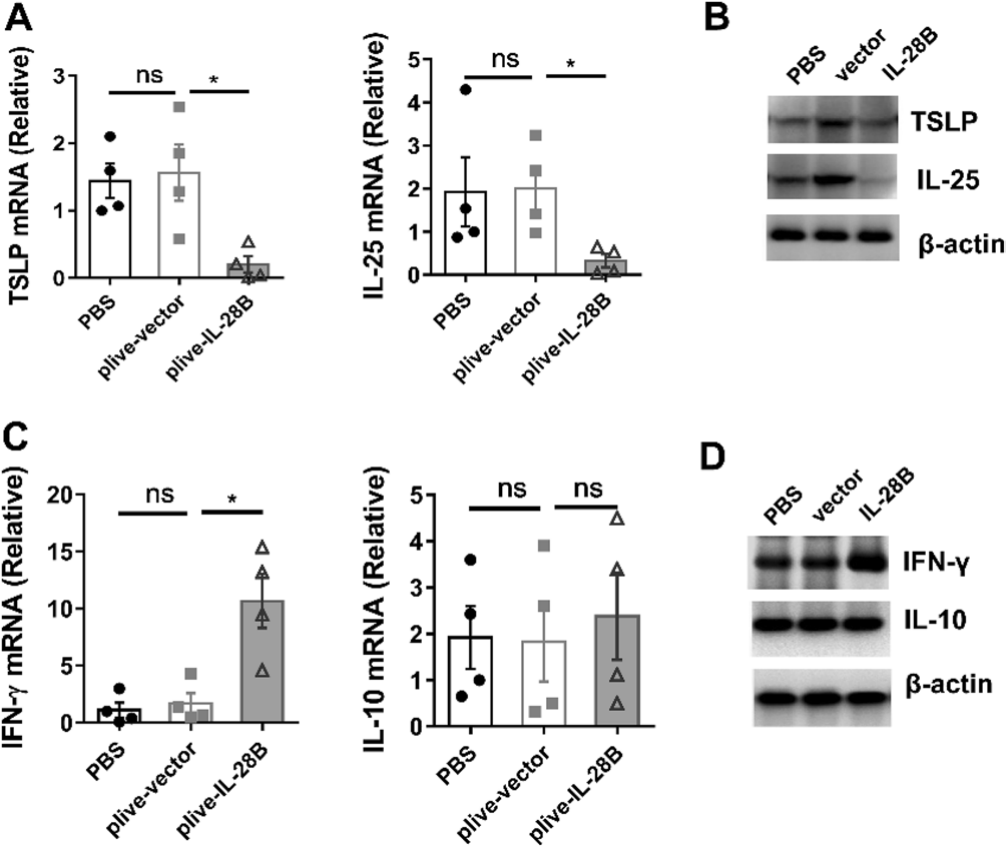
IL-28B treatment reduced production of IL-25 and TSLP after papain challenged. Mice were treated as previously and lung tissues were harvested on day 7. (**A**) The total mRNA of lung tissues was extracted and the mRNA levels of TSLP and IL-25 were determined by Real-time PCR. (**B**) The protein levels of TSLP (~ 15.4 kDa) and IL-25 (~ 20 kDa) in lung tissues were determined by Western Blot. (**C**) The mRNA levels of IFN-γ and IL-10 in lung tissues were determined by Real-time PCR. (**D**) The protein levels of IFN-γ (~ 15.6 kDa) and IL-10 (~ 18.7 kDa) in lung tissues were determined by Western Blot. Data are representative of three or more independent experiments. Data are the mean ± SEM. *P < 0.05, ns = not significant.

To ascertain which signaling pathway was involved in the protective function of IL-28B in this model, we detected expression pattern of some molecules, including both anti-asthmatic and pro-asthmatic candidates. The reduction of not only eosinophils but also neutrophils in mice with IL-28B treatment before papain challenge reminder that IL-28B might affect the upstream events such as activation of alarmin. Indeed, the expressions of alarmin including TSLP and IL-25 were significantly down-regulated in both of transcript and protein levels from lung tissues of plive-IL-28B treated mice when compared with the counterparts from PBS or plive-vector treated mice (Fig. 3A,B, Supplementary Fig. 2A and B). Moreover, the level of IFN-γ but not IL-10 was up-regulated after plive-IL-28B treatment, which were all anti-asthmatic players in previous reports (Fig. 3C,D, Supplementary Fig. 2C and D). These data indicated the protective function of IL-28B might ascribe to TSLP and IL-25 signal pathway.

### IL-28B regulates epithelial cells response to papain challenge

**Figure 4:**
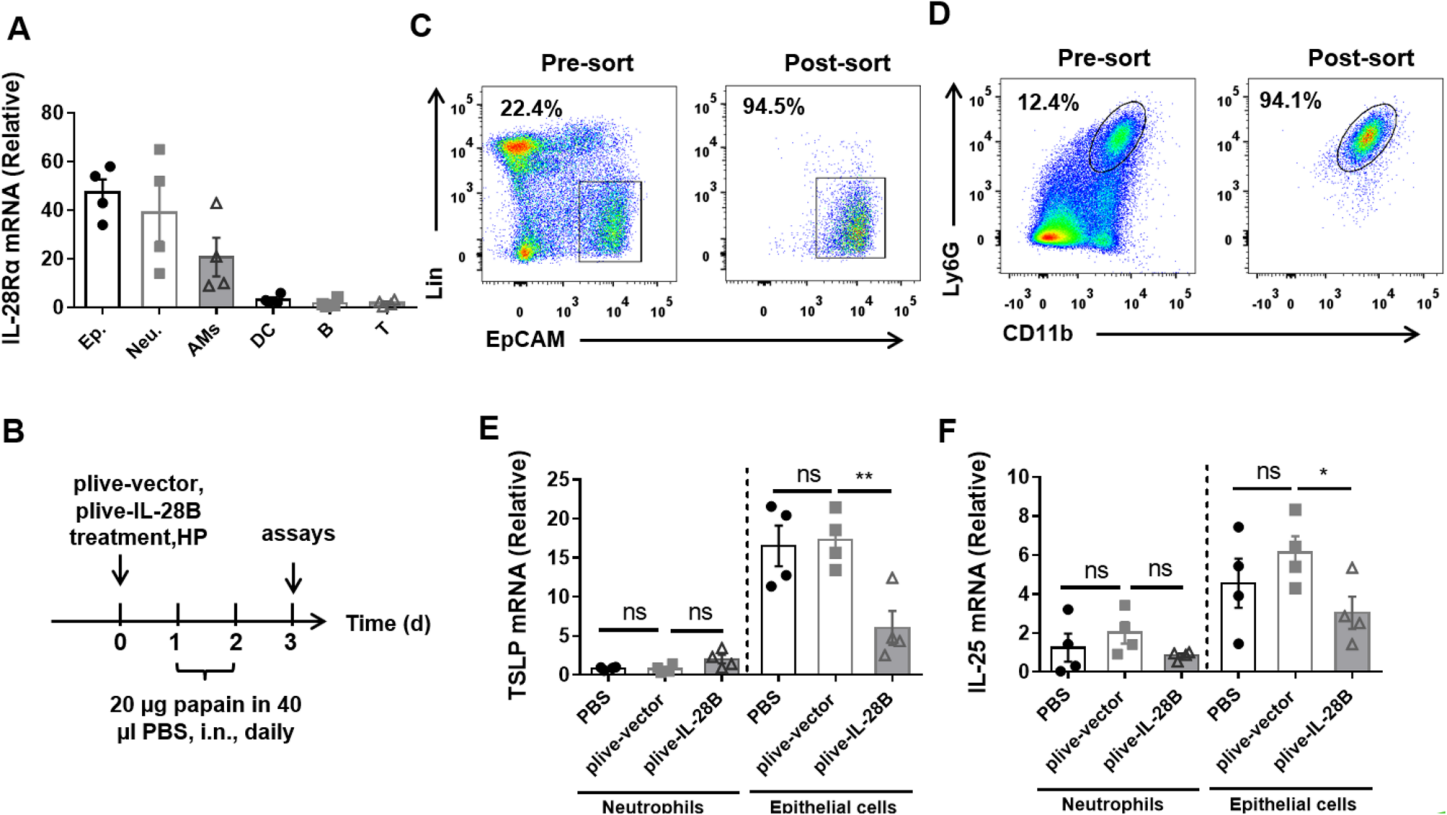
Epithelial cells but not neutrophils responsed to IL-28B signal in papain-induced asthma. (**A**) Lung tissues from naïve WT mice were prepared and epithelial cells, neutrophils, AMs, DC, T and B cells were isolated, respectively. 0.1 million cells were used for mRNA extraction. And the mRNA levels of IL-28Rα (*IFNLR1*) were analyzed by Real-time PCR. (**B**) Work flow for IL-28B treatment in papain-induced asthmatic mice. Mice were injected (h.p.) with plive-IL-28B or plive-vector or PBS on day 0 and were subsequently challenged (i.n.) with papain for 2 consecutive days. (**C**) The lung tissues were harvested on day 3 and the lung cells were prepared. The epithelial cells (Lin^−^EpCAM^+^, Lin = CD45, CD16/32, CD31, Ter119, and integrin β4) were sorted by FACS. The purity after sorting was shown. (**D**) The neutrophils (CD45^+^CD11b^+^Ly6G^+^) from lung tissues on day 3 were sorted by FACS. The purity after sorting was shown. (**E–F**) At day 3, lung cells were prepared and neutrophils and epithelial cells were sorted as shown in (**C**) and (**D**). 0.1 million cells were used for mRNA extraction and mRNA levels of TSLP (**E**) and IL-25 (**F**) were analyzed by Real-time PCR. Data are representative of three or more independent experiments. Data are the mean ± SEM. *P < 0.05, **P < 0.01, ns = not significant. h.p., hydrodynamic injection; i.n., intranasally injection.

TSLP and IL-25 are important initiators in both human asthmatic patients and mouse models. When host encounters the allergen, TSLP and IL-25 are produced by different types of cells quickly, largely by first line of host cells like epithelial cells, macrophages, DCs and neutrophils. This may happen in hours to several days. To check which cell could be modulated by IL-28B and if it functions in a direct or indirect way, we analyzed the IL-28Ra (*IFNLR1*) expression pattern in these cells. The results show that IL-28Ra is highly expressed on epithelial cells and neutrophils, and also on alveolar macrophages at a lower level, but rarely on DC, T cells and B cells (Fig. 4A). We treated mice plive-IL-28B or plive-vector or PBS followed by two times of papain challenges and harvested lung tissue for cells sorting (Fig. 4B–D). We found that, after papain challenge, TSLP and IL-25 highly expressed on epithelial cells but not neutrophils. Moreover, the levels of TSLP and IL-25 from epithelial cells but not neutrophils dramatically were down-regulated in plive-IL-28B treated mice compared with plive-vector or PBS treated mice (Fig. 4E,F). Collectively, these data indicated that IL-28B regulates epithelial cells response to papain challenge in a direct way.

### IL-28B regulates IL-25 and TSLP expression in epithelial cells via Erk

**Figure 5:**
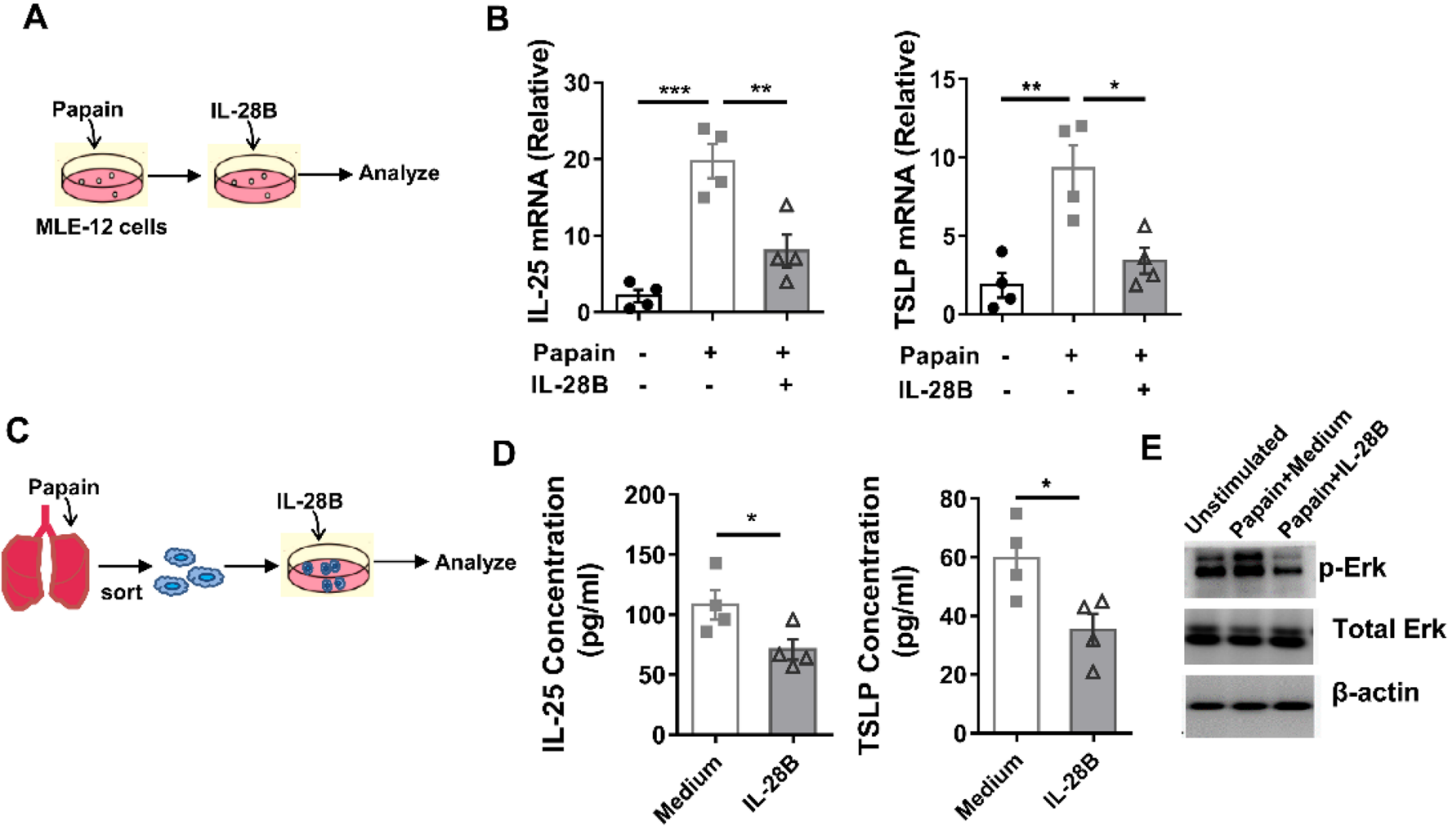
IL-28B down-regulated IL-25 and TSLP expression in epithelial cells by targeting Erk. (**A**) Schematic diagram for in vitro culture: MLE-12 cells were treated with 200 ng/ml papain. After 4 h, IL-28B (100 ng/ml) was added and cocultured for 24 h. (**B**) The mRNA levels of TSLP and IL-25 in MLE-12 cells were determined by Real-time PCR. (**C**) Schematic diagram for ex vivo culture: Mice were treated with 20 μg of papain (i.n.) for two times, once a day. One day last challenge, epithelial cells were sorted and cultured with 200 ng/ml papain. After 2 h, IL-28B (100 ng/ml) was added and cocultured for 24 h. (**D**) The concentration of IL-25 and TSLP in supernatant were determined by ELISA. (**E**) The phosphorylation of Erk (~ 42.4 kDa) and total Erk (~ 42.4 kDa) in epithelial cells were analyzed by Western Blot. Data are representative of three or more independent experiments. Data are the mean ± SEM. *P < 0.05, **P < 0.01, ***P < 0.001. i.n., intranasally injection.

Next, we explore if IL-28B affect epithelial cells in a direct and which signal pathway were involved. We employ an in vitro culture system with MLE-12 cell line that is a epithelial cell line of lung alveolar type II epithelial cells (Fig. 5A). MLE-12 cells were able to produce high amount of IL-25 and TSLP after papain stimulation in vitro, as indicated by the transcript level (Fig. 5B). Moreover, IL-25 and TSLP were largely inhibited after IL-28B cytokine added. (Fig. 5B). This phenotype was also seen in primary lung epithelial cells from papain challenge mice ex vivo (Fig. 5C,D), suggesting that IL-28B directly down-regulated IL-25 and TSLP production in this process. To further investigate the intracellular mechanism that how IL-28B and its receptor interaction influence the IL-25 and TSLP producing by epithelial cells, we analyze the converge of the two pathways. We found that the phosphorylation of Erk in epithelial cells was significantly down-regulated after IL-28B treatment (Fig. 5E and Supplementary Fig. 3). These data indicate that IL-28B regulates IL-25 and TSLP expression in epithelial cells by inhibition of Erk.

## Discussion

IFN-λ was originally known to have antiviral and antitumor activities^17–23^. More recently, the regulation property of this cytokine in asthma has attract attentions^24,25^. It has been reported that IL-28A and IL-28B suppressed allergic airway disease through modulating lung DC function and NK cell function, respectively^13,14^. In fact, their receptors are highly expressed on epithelial cells (Fig. 4A). Interestingly, the epithelial cells of airways serve as the first line of defense against inhaled allergens. But if IL-28-IL-28R take part in regulation of epithelial cells response to allergens remain unclear. In this paper, we demonstrated that activation of IL-28R by IL-28B could reduce papain-induced airway hyper-responsiveness (Fig. 1) and inflammatory immune response (Fig. 2), which is associated with down-regulation of phosphorylation of Erk signaling pathway (Fig. 5) and decreased production of IL-25 and TSLP.

To our knowledge, asthma is the consequence of interaction among environmental factors, host immune cells and non-immune cells. Epithelial cells of the airway form a physical barrier against the external environmental factor and disruption of epithelial barrier initiate type 2 immune responses^26,27^. Proteases such as papain from plant or Der p1 from HDM stimulate epithelial cells to secrete the cytokines TSLP, IL-25, and IL-33, which act on subepithelial dendritic cells and innate lymphoid cells to initiate the release of Th2 cytokines^28^. This crosstalk is utmost important for pathogenesis of asthma. Indeed, our results showed that high amount of IL-25 and IL-33 were detected after papain challenge (Fig. 3A,B). Notably, IL-28B treatment significantly decreased the production of both of the cytokines (Fig. 4E,F). It is interesting to know whether this signaling pathway also works in other mouse models such as OVA induced asthma or HDM induced asthma or in human. We suppose this should have overlap with HDM model as HDM contains protease Der P1 and Der P5^29^. Another study reported inhaled delivery of IL-28 restricts epithelial-derived Th2 inflammation in OVA induced asthma^25^, which is consistent with our results.

To explore the role of IL-28B in papain-induced airway hyper-responsiveness, we used a supraphysiological IL-28B–overexpressing mice model, in which IL-28B is overexpressed systemically. Even though it is an artificial manipulation, hydrodynamic injection of IL-28B vector is able to produce active protein stably for at least 2 weeks. Mice administrated with IL-28B through hydrodynamic injection were relatively normal because IL-28R is expressed on limited cell types. So it is useful to study the function of IL-28B and reflects the situation under the pathological states in vivo to some extent. And its function was demonstrated by the experiments in vitro.

Previous studies showed that IL-28B also affected NK cell function in virus infection and OVA-induced asthma^14,16^. It is not clear if IL-28B-NK axis also involve in papain-induced asthma. The epithelial cells of airways serve as the first line of defense against inhaled allergens. Notably, epithelial cells express high level of IL-28B receptor. Here, we highlight the IL-28B-epithelial cells axis as epithelial cells are the prominent cells responsive to IL-28B.

IL-28B belongs to type III IFN. IL-28B shows potential advantage in use when compared with Type I IFN as Type I IFN showed severe side effects in clinical trials^30^. In our study, mice administrated with IL-28B through hydrodynamic injection were relatively normal because IL-28R is expressed on limited cell types. Furthermore, epithelial cell dysfunction acts as a major driver of asthma development, mainly because improper activation and production of IL-25 and TSLP from epithelial cells trigger allergy response. Our data reveal that IL-28B directly modulate IL-25 and TSLP signaling pathway in epithelial cells. Therefore, IL-28B may serve as a potential therapeutic agent for asthma disease.

## Supplementary information

Supplementary Figures.
