# Supplementary material for: Interleukin-28B dampens protease-induced lung inflammation via IL-25 and TSLP inhibition in epithelial cells
Source: Sci Rep. 2020 Dec 1;10:20973. doi: 10.1038/s41598-020-77844-y (PMC7708501; doi:10.1038/s41598-020-77844-y)
Supplement: Supplementary file 1 — Supplementary Figures. [file 41598_2020_77844_MOESM1_ESM.pdf]

Interleukin-28B dampens protease-induced lung inflammation via IL-25 and TSLP inhibition in epithelial cells

Bailing Yan[1], Jinying Gao[1], Jia Guo[1], Dong Yang[2], Dan Li[1]

[1]Department of Respiratory Medicine, The First Hospital of Jilin University, 1Xinmin Street, Changchun, 130021, Jilin Province, People's Republic of China

[2]Department of Gastroenterology, The First Hospital of Jilin University, 1Xinmin Street, Changchun, 130021, Jilin Province, People's Republic of China

Corresponding author: Dan Li: li_dan@jlu.edu.cn

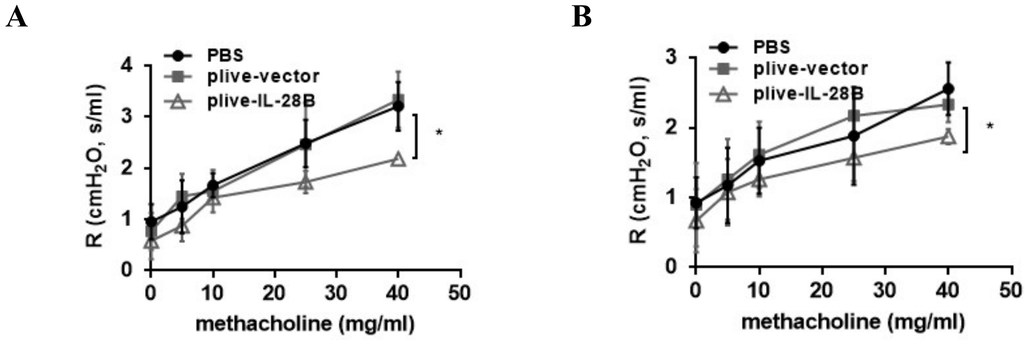

Supplementary Figure 1. Airway resistance was measured in response to increasing doses of aerosolized methacholine in mice (two-way ANOVA comparing plive-vector-treated and plive-IL-28B-treated mice). **A** and **B** represent the results of two independent experiments with 4 mice per group. Data are the mean ± SEM. **P* < 0.05. (**Related to Figure 1E)**

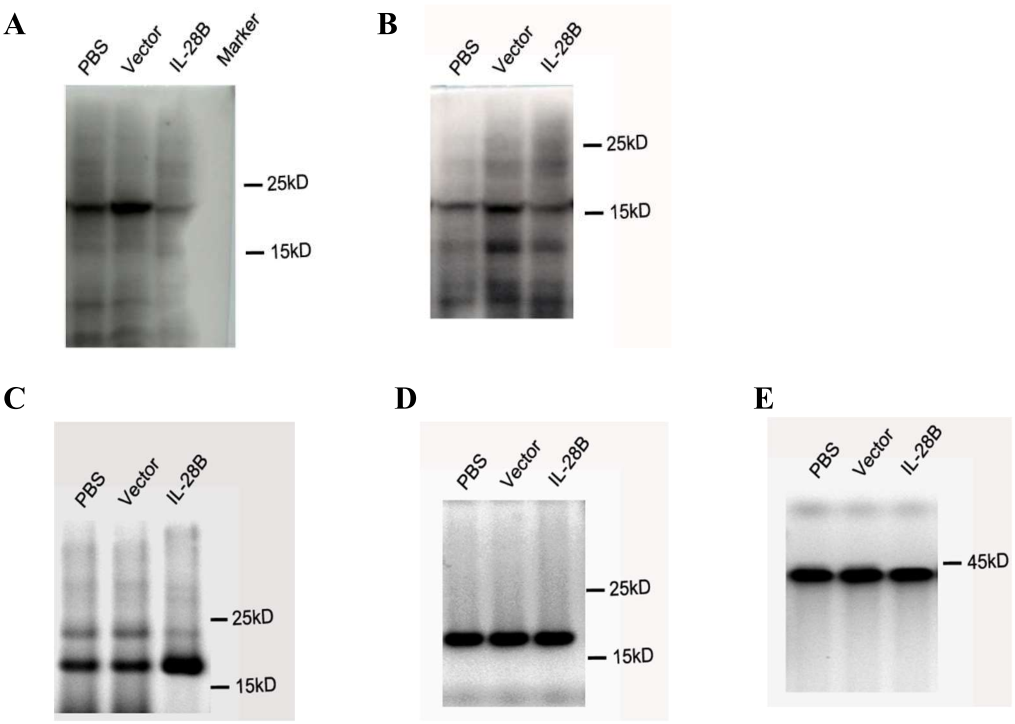

Supplementary Figure 2. Western Blot analysis of protein levels of IL-25(**A**) and TSLP(**B**) in lung tissues **(Related to Figure 3B)**. Western Blot analysis of protein levels of IFN-γ(**C**) and IL-10(**D**) in lung tissues **(Related to Figure 3D)**. β-actin(**E**) was analyzed as housekeeping protein.

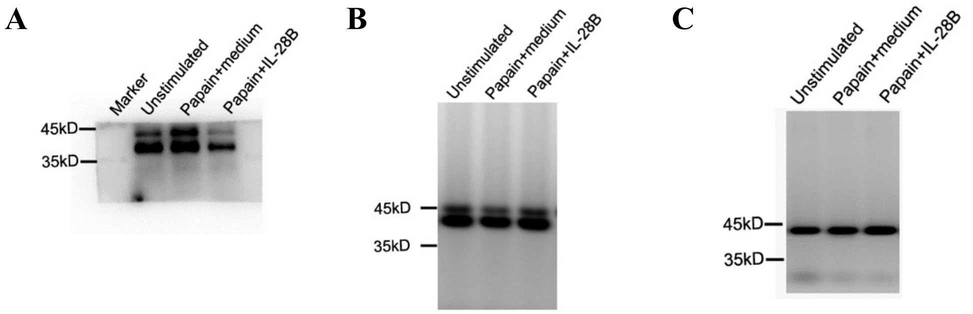

Supplementary Figure 3. The phosphorylation of Erk(**A**) and total Erk (**B**) in epithelial cells were analyzed by Western Blot. β-actin(**C**) was analyzed as housekeeping protein(**Related to Figure 5E**).
